# Effect of Ultrasound-Guided Transversus Abdominis Plane Block Combined with Patient-Controlled Intravenous Analgesia on Postoperative Analgesia After Laparoscopic Cholecystectomy: a Double-Blind, Randomized Controlled Trial

**DOI:** 10.1007/s11605-022-05450-6

**Published:** 2022-09-13

**Authors:** Liming Dai, Xiangwei Ling, Yuying Qian

**Affiliations:** Department of Anesthesiology, The Second Affiliated Hospital of Wannan Medical College, No.123 Kangfu Road, Jinghu District, Wuhu, 241000 Anhui China

**Keywords:** Laparoscopic cholecystectomy, Postoperative pain, Transversus abdominis plane block, Patient-controlled intravenous analgesia, Ultrasound

## Abstract

**Purpose:**

To compare the effect of ultrasound-guided transversus abdominis plane block (TAPB) combined with patient-controlled intravenous analgesia (PCIA) and PCIA alone on analgesia after laparoscopic cholecystectomy (LC).

**Methods:**

In this double-blind, randomized controlled trial, 160 patients undergoing LC were randomized into the TAPB group (*n* = 80) and PCIA group (*n* = 80). Bilateral ultrasound-guided TAPB was performed with 20 mL 0.5% ropivacaine and the PCIA pump was given after LC in the TAPB group. The PCIA group received the PCIA pump alone as a control group. The primary outcome was postoperative pain, assessed by the visual analog scale (VAS).

**Results:**

VAS pain (including abdominal wall pain or visceral pain) scores at rest and coughing were significantly lower in the TAPB group at 1, 4, 12, 24, 36, and 48 h after LC (*P* < 0.05). Postoperative additional analgesic needs, analgesic pump compressions, and PCIA analgesic dosages, and total morphine equivalents were significantly reduced in the TAPB group, and postoperative hospital stay, total hospitalization expenses, expenses within 24 h or 48 h (from analgesia and adverse reactions), and patient satisfaction were significantly higher in the TAPB group than the PCIA group (all *P* < 0.05). No significant between-group differences were observed in operation time, intraoperative blood loss, unplugging the analgesic pump due to adverse reactions, first exhaust time, and postoperative adverse events between the two groups.

**Conclusions:**

Ultrasound-guided TAPB combined with PCIA was an effective and safe perioperative analgesic technique for patients undergoing LC compared to PCIA only.

**Supplementary Information:**

The online version contains supplementary material available at 10.1007/s11605-022-05450-6.

## Introduction

Laparoscopic cholecystectomy (LC) is a frequently performed minimally invasive procedure that causes less perioperative pain than open procedures. However, laparoscopic surgery can result in moderate to severe postoperative pain.^1,2^ Patient-controlled intravenous analgesia (PCIA) with opioids and non-steroidal anti-inflammatory drugs (NSAIDs) is the most common analgesic technique. However, it is associated with many side effects related to opioids, such as respiratory depression, itching, urinary retention, and weak pharyngeal musculature (hence breathing problems).^3–5^ As an alternative to opioid-based PCIA, transversus abdominis plane block (TAPB), which involves the blockade of sensory nerve signals to the anterolateral abdominal wall, can produce long-lasting analgesia after abdominal surgery.^6^ Improvements have been made to TAPB with ultrasound guidance to confirm the proper region and avoid complications through direct needle visualization.^7,8^ Combined analgesic regimens or multimodal approaches have been recommended in managing postoperative pain, minimizing the dose of medications and reducing adverse effects while still providing adequate analgesia.^9–11^

As a newer technique, ultrasound-guided TAPB is more manageable and perhaps safer as part of multimodal analgesia. Although the use of TAPB has become more widespread in LC, studies comparing the analgesic efficacy of TAPB are very few with conflicting results. Previous studies reported that TAPB improved postoperative analgesia in patients undergoing LC,^12^ resulting in lower visual analog scale (VAS) scores and total analgesic consumption.^13,14^ However, several studies showed that TAPB had no benefit in reducing opioid consumption and pain after LC in patients provided with multimodal analgesia.^15,16^ Compared to PCIA with or without single-shot TAPB, ultrasound-guided continuous TAPB provided similar analgesia in somatic pain and less analgesia in visceral pain.^15^ Therefore, further research should be undertaken to investigate the value of TAPB during pain management after LC.

In this double-blind, randomized controlled trial (RCT), we assessed the efficacy, safety, and cost of ultrasound-guided TAPB combined with PCIA versus simple PCIA for decreasing postoperative pain in patients undergoing LC, which may provide a reference for clinical practice.

## Materials and Methods

This double-blind RCT was conducted at the Second Affiliated Hospital of Wannan Medical College between June 2020 and January 2021. This study obtained approval from the Institutional Ethical Review Board (approval number: WYEFYLS202008), and was registered with Clinical Trials (registration number: ChiCTR2000034001). The study protocol conformed to the ethics guidelines of the Declaration of Helsinki. Written informed consents were obtained from all participants before surgery.

### Participants

Inclusion criteria: age ≥ 18 years old; patients scheduled to undergo laparoscopic cholecystectomy and requiring postoperative analgesia; the American Society of Anesthesiologists (ASA) physical status I-II; no communication barriers, able to cooperate in the implementation of interventions and understand the use of related scales, and able to operate PCIA equipment.

Exclusion criteria: having contraindications to nerve block (such as infection at the puncture site); metastatic tumor indicated by preoperative examination; suffering from severe liver and kidney disease, coagulation dysfunction, etc.; previous history of abdominal surgery or trauma; long-term use of sedative and analgesic drugs, or addiction to alcohol, sedative and analgesic drugs; undergoing chronic pain; allergic to the drugs used in the study; suffering from mental illness that interferes with perception and pain assessment; taking analgesics before surgery; women who were pregnant or breastfeeding; participating in other clinical studies and had participated in other clinical studies within 30 days; other situations that the investigators considered inappropriate to participate in the study.

### Randomization and Blinding

According to a computer-generated random number table with a 1:1 allocation, patients were randomly assigned into two groups before the start of surgery: PCIA group and TAPB group. Sequentially numbered, opaque, sealed envelopes were prepared, and provided group assignment details and case numbers. The patients in the PCIA group (*n* = 80) received the PCIA pump alone as a control group. The patients in the TAPB group (*n* = 80) received ultrasound-guided TAPB with 0.5% ropivacaine (20 mL) and the PCIA pump. Both the patients and efficacy evaluators were blinded to the group assignment.

### Analgesic Technique

In the operating room (OR), standard monitoring with electrocardiography, noninvasive blood pressure, peripheral oxygen saturation, and heart rate were applied to all the patients. Induction of anesthesia was performed by injecting midazolam (0.05 mg/kg), sufentanil (0.03 μg/kg), etomidate (0.3 mg/kg), and cisatracurium (0.15 mg/kg). Tracheal intubation and mechanical ventilation were performed after good induction of anesthesia. Intravenous propofol (6 mg/kg/h) and remifentanil (0.3 μg/kg/min) were infused and titrated for anesthesia maintenance.

After surgery, when vital signs were stable, bilateral TAPB was performed under ultrasonographic guidance by the same anesthetist involved in the study. First, identify and mark the Petit triangle and routinely disinfect the skin on both sides of the abdominal wall. Anatomical landmarks were recognized at the level of the anterior axillary line between the iliac crest and the costal margin. The block was performed using a 22-gauge, 100-mm nerve blockade needle, and an injection line was visualized using an “in-plane” ultrasound-guided technique. Once the tip of the needle was placed in the space between the rectus abdominis muscle and internal oblique abdominal muscle, and negative aspiration was confirmed, 20 mL of 0.5% ropivacaine was administered under direct ultrasound guidance. Likewise, TAPB was performed on the contralateral side. The PCIA group did not receive TAPB.

For postoperative pain management, both the TAPB group and PCIA group received the PCIA pump with 100 mL of normal saline mixed with sufentanil (100 μg) and dezocine (20 mg). The basal infusion rate was set to 2 mL/h, the bolus dose was 0.5 mL, and a lockout interval of 15 min was maintained for 48 h postoperatively.

### Data Collection

Before the operation, data on age, sex, nationality, height, weight, ASA classification, comorbidity, indication for LC, and type of surgery (elective, emergent) of the patients were collected. During the perioperative period, the severity of pain (abdominal wall pain and visceral pain) at rest and while coughing was evaluated using the VAS score 1, 4, 12, 24, 36, and 48 h after surgery. The Ramsay Sedation Scale (RSS) score (1 = anxious and agitated or restless or both, 2 = cooperative, oriented, and tranquil, 3 = responding to commands only, 4 = exhibiting a brisk response to a light glabellar tap, 5 = exhibiting a sluggish response to a light glabellar tap, and 6 = exhibiting no response)^17^ was recorded 1, 4, 12, 24, 36, and 48 h after surgery. Patient satisfaction with postoperative analgesia was assessed using a 4-point Likert scale (1 = very dissatisfied, 2 = somewhat dissatisfied, 3 = somewhat satisfied, and 4 = very satisfied).^18^ Information about operation time, intraoperative blood loss, postoperative hospital stay, number of analgesic pump compressions within 24 h, PCIA analgesic dosages within 24 h, unplugging the analgesic pump due to adverse reactions, postoperative additional analgesic needs, first leaving bed time, first exhaust time, concomitant medication, total hospitalization expenses, and expenses within 24 h and 48 h (analgesia and adverse reactions) were obtained. Intravenous 100 mL sufentanil (0.1 μg/kg) was used for postoperative additional analgesia. Total morphine equivalents were calculated considering 1 μg intravenous sufentanil = 3 mg oral morphine and 1 mg intravenous dezocine = 3 mg oral morphine. The incidences of postoperative adverse events (hypotension, bradycardia, respiratory depression, nausea or vomiting, itching, infection) were also recorded.

### Outcome Measures

The study’s primary outcome was the efficacy of postoperative analgesia between the two groups by comparing VAS in the first 48 postoperative hours at the mentioned time points. The efficacy was measured by abdominal wall pain and visceral pain scores at rest and while coughing. Secondary outcomes were operation time, intraoperative blood loss, postoperative hospital stay, patient satisfaction with postoperative analgesia, number of analgesic pump compressions within 24 h, PCIA analgesic dosages within 24 h, unplugging the analgesic pump due to adverse reactions, postoperative additional analgesic needs, first leaving bed time, first exhaust time, total hospitalization expenses, and expenses within 24 and 48 h (from analgesia and adverse reactions), postoperative adverse events, and concomitant medication.

### Cost-effectiveness Analysis

The mean reduction in the VAS score during the 48-h study duration was calculated, using an unweighted average to combine the six measurements (at 1, 4, 12, 24, 36, and 48 h) into one summary value. For the cost-effectiveness analysis, the reduction in pain intensity was multiplied by the duration of the study (2 days) to obtain the change in “VAS-days.’’ The incremental cost-effectiveness ratio (ICER) was calculated by the usual formula: ICER = (Costs _TAPB group_- Costs _PCIA group_)/(Pain _TAPB group_- Pain _PCIA group_). This ratio estimated the additional cost of achieving a one point change in the VAS-days score for pain.

### Statistical Analysis

Quantitative data were tested by Kolmogorov–Smirnov for normality. Normally distributed measurement data were described as mean ± standard deviation (mean ± SD), and comparison between the two groups was performed by the independent-sample *t*-test. Non-normal data were expressed by median and interquartile range [M (Q_1_, Q_3_)], and the Mann–Whitney *U* rank sum test was used for comparison between the groups. Enumeration data were shown as the number of cases and constituent ratio [n (%)], inter-group comparison was conducted with the chi-square test, and the rank sum test was applied for ranked data. All statistical tests were two-sided, and the test level was α = 0.05. SAS version 9.4 (SAS Institute, Cary, NC, USA) was utilized for difference analysis, stacked graphs and histograms were drawn using GraphPad Prism version 8 (GraphPad Software, San Diego, California, USA), and box plots were developed with R 4.20 (R Foundation for Statistical Computing, Vienna, Austria).

## Results

### Characteristics of the Included Patients

**Fig. 1:**
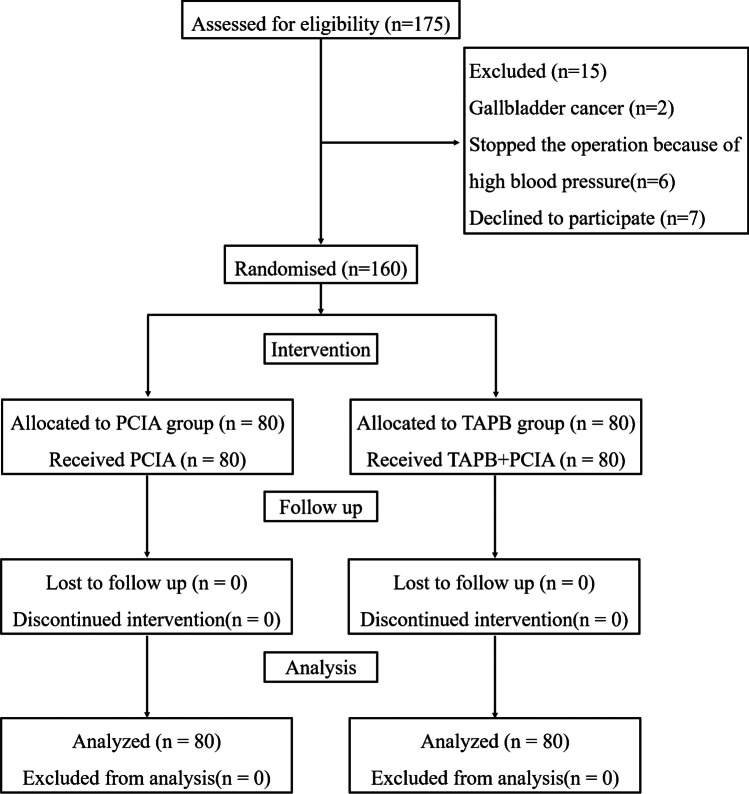
Flowchart of participant selection. PCIA group: the patients received the PCIA pump alone, TAPB group: the patients received ultrasound-guided TAPB combined with the PCIA pump, PCIA: patient-controlled intravenous analgesia, TAPB: transversus abdominis plane block

Among 175 eligible patients, 7 patients refused to be included in the study, 2 were excluded due to gallbladder cancer, and 6 patients stopped the operation because of high blood pressure (Fig. 1). In the end, 160 patients were included for analysis, randomly assigned to the TAPB and PCIA groups. No statistically significant differences concerning sex, age, body mass index (BMI), nationality, ASA physical status, comorbidity, and indication for LC were found between the groups (all *P* > 0.05). All the patients underwent elective surgery. Patient characteristics are shown in Table 1.

**Table 1:** Characteristics of the included patients in the PCIA and TAPB groups

Variable	Total (*n* = 160)	PCIA group (*n* = 80)	TAPB group (*n* = 80)	*P*
Gender, n (%)				0.230
Male	49 (30.6)	28 (35.0)	21 (26.3)	
Female	111 (69.4)	52 (65.0)	59 (73.8)	
Age (years), Mean ± SD	56.3 ± 14.4	55.3 ± 14.0	57.2 ± 14.8	0.418
BMI (kg/m^2^), Mean ± SD	24.3 ± 3.4	23.9 ± 2.7	24.7 ± 4.0	0.136
Nationality, n (%)				1.000
Han	157 (98.1)	78 (97.5)	79 (98.8)	
Other	3 (1.9)	2 (2.5)	1 (1.3)	
ASA, n (%)				1.000
I	118 (73.8)	59 (73.8)	59 (73.8)	
II	42 (26.3)	21 (26.3)	21 (26.3)	
Combined with other diseases, n (%)				0.715
No	120 (75.0)	61 (76.3)	59 (73.8)	
Yes	40 (25.0)	19 (23.8)	21 (26.3)	
Indication for LC, n (%)				0.385
Gallstone	67 (41.9)	31 (38.8)	36 (45.0)	
Chronic acalculous cholecystitis	45 (28.1)	21 (26.3)	24 (30.0)	
Chronic calculous cholecystitis	48 (30.0)	28 (35.0)	20 (25.0)	

PCIA group: the patients received the PCIA pump alone, TAPB group: the patients received ultrasound-guided TAPB combined with the PCIA pump, *PCIA* patient-controlled intravenous analgesia, *TAPB* transversus abdominis plane block, *SD* standard deviation, *BMI* body mass index, *ASA* American Society of Anesthesiologist, *LC* laparoscopic cholecystectomy

### Efficacy and Cost of Ultrasound-Guided TAPB Combined with PCIA

**Fig. 2:**
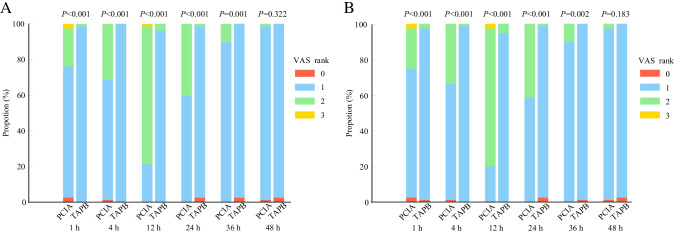
Postoperative VAS scores of abdominal wall pain (**A**) or visceral pain (**B**) at rest at various time points. Values are in the number of patients (percentage) with Visual Analogue Scale (VAS) scores 0/1/2/3 (0 = no pain, 1 = mild pain, 2 = moderate pain, 3 = severe pain). PCIA group: the patients received the PCIA pump alone, TAPB group: the patients received ultrasound-guided TAPB combined with the PCIA pump, PCIA: patient-controlled intravenous analgesia, TAPB: transversus abdominis plane block

**Fig. 3:**
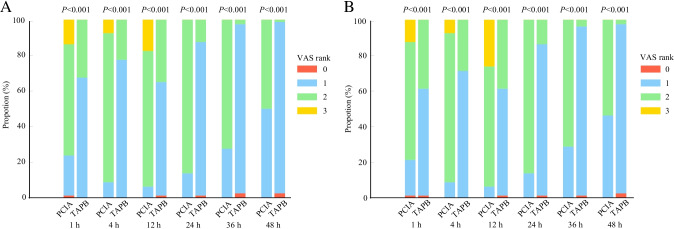
Postoperative VAS scores of abdominal wall pain (**A**) or visceral pain (**B**) while coughing at various time points. Values are in the number of patients (percentage) with Visual Analogue Scale (VAS) scores 0/1/2/3 (0 = no pain, 1 = mild pain, 2 = moderate pain, 3 = severe pain). PCIA group: the patients received the PCIA pump alone, TAPB group: the patients received ultrasound-guided TAPB combined with the PCIA pump, PCIA: patient-controlled intravenous analgesia, TAPB: transversus abdominis plane block

The effectiveness of analgesic techniques was assessed by the VAS at specific time points during 48 h at rest and while coughing. As shown in Supplementary Table 1, more patients had VAS scores of 1 in the TAPB group versus the PCIA group for abdominal wall pain (98.8 vs. 73.8%) and visceral pain (96.3 vs. 72.5%) at rest as well as abdominal wall pain (67.5 vs. 22.5%) and visceral pain (60.0 vs. 20.0%) while coughing, and less patients had a RSS score of 1 in the TAPB group versus the PCIA group (2.5 vs. 27.9%) at 1 h after LC. Significant differences were found in these VAS scores and RSS scores at 1 h after LC between the TAPB and PCIA groups (all *P* < 0.05). At 4 h after LC, more patients had VAS scores of 1 in the TAPB group versus the PCIA group for abdominal wall pain (100.0 vs. 67.5%) and visceral pain (98.8 vs. 65.0%) at rest as well as abdominal wall pain (77.5 vs. 8.8%) and visceral pain (71.3 vs. 8.8%) while coughing, and VAS scores for abdominal wall pain and visceral pain both at rest and while coughing in the TAPB group were significantly lower than those in the PCIA group (all *P* < 0.05) (Supplementary Table 2). At 12 h after LC, the TAPB group had significantly decreased VAS scores for abdominal wall pain and visceral pain both at rest and while coughing compared with the PCIA group (all *P* < 0.05) (Supplementary Table 3). At 24 h after LC, more patients had VAS scores of 0 and 1 in the TAPB group versus the PCIA group for abdominal wall pain (98.8 vs. 60%) and visceral pain (98.8 vs. 58.8%) at rest as well as abdominal wall pain (87.6 vs. 13.8%) and visceral pain (86.3 vs. 13.8%) while coughing, and these VAS scores in the TAPB group were significantly lower than those in the PCIA group (all *P* < 0.05) (Supplementary Table 4). Similar to the pain scores at 24 h after LC, VAS scores at 36 h and 48 h after LC in the TAPB group were significantly reduced in contrast to those in the PCIA group (all *P* < 0.05) (Supplementary Tables 5 and 6). Patients in the TAPB group had significantly lower VAS scores for abdominal wall pain and visceral pain at rest than the patients in the PCIA group at all time points except 48 h (Fig. 2A, B). Comparisons of VAS scores (for abdominal wall pain and visceral pain) while coughing demonstrated significantly higher values in the PCIA group at all time points (all *P* < 0.05) (Fig. 3A, B). The postoperative RSS score at all time points except one hour after LC did not differ significantly between the two groups (all *P* > 0.05) (Supplementary Fig. 1).

Other indicators in the perioperative period were also evaluated. We found that first leaving bed time, number of analgesic pump compressions within 24 h, and PCIA analgesic dosages within 24 h were significantly lower in the TAPB group than those in the PCIA group (all *P* < 0.05) (Supplementary Figs. 2A-C). Compared with the TAPB group, the PCIA group had significantly greater postoperative additional analgesic needs (*P* < 0.05) (Supplementary Fig. 2D). The total morphine equivalents of the TAPB group were significantly lower than those of the PCIA group (364.9 ± 9.0 mg vs. 369.5 ± 9.5 mg) (*P* < 0.05). Postoperative hospital stay, total hospitalization expenses, and expenses within 24 and 48 h (from analgesia and adverse reactions) were significantly higher in the TAPB group than in the PCIA group (all *P* < 0.05) (Supplementary Figs. 2E-H). Additionally, patients receiving TAPB after LC were more satisfied than those in the PCIA group (*P* < 0.05) (Supplementary Fig. 2I). No significant intergroup differences were found in operation time, intraoperative blood loss, unplugging the analgesic pump due to adverse reactions, and first exhaust time (all *P* > 0.05) (Table 2).

**Table 2:** Other indicators in the perioperative period

Variable	Total (*n* = 160)	PCIA group (*n* = 80)	TAPB group (*n* = 80)	*P*
Operation time (min), M (Q_1_, Q_3_)	45.0 (37.0, 56.0)	45.0 (37.0, 54.0)	48.5 (36.0, 61.5)	0.381
Intraoperative blood loss, n (%)				1.000
No	1 (0.6)	1 (1.3)	0 (0.0)	
Yes	159 (99.4)	79 (98.8)	80 (100.0)	
Postoperative hospital stay (days), M (Q_1_, Q_3_)	4.0 (3.0, 5.0)	3.0 (3.0, 4.0)	4.0 (4.0, 5.0)	< 0.001
Patient satisfaction with postoperative analgesia, n (%)				< 0.001
1 = very dissatisfied	10 (6.3)	10 (12.5)	0 (0.0)	
2 = somewhat dissatisfied	27 (16.9)	27 (33.8)	0 (0.0)	
3 = somewhat satisfied	50 (31.3)	28 (35.0)	22 (27.5)	
4 = very satisfied	73 (45.6)	15 (18.8)	58 (72.5)	
Number of analgesic pump compressions within 24 h, M (Q_1_, Q_3_)	1.0 (0.0, 3.0)	3.0 (2.0, 4.0)	0.0 (0.0, 0.0)	< 0.001
PCIA analgesic dosages within 24 h (mL), Mean ± SD	57.9 ± 8.3	64.7 ± 6.1	51.2 ± 3.1	< 0.001
Unplugging the analgesic pump due to adverse reactions, n (%)				0.276
No	152 (95.0)	78 (97.5)	74 (92.5)	
Yes	8 (5.0)	2 (2.5)	6 (7.5)	
Postoperative additional analgesic needs, n (%)				< 0.001
No	100 (62.5)	39 (48.8)	61 (76.3)	
Yes	60 (37.5)	41 (76.3)	19 (23.8)	
Total morphine equivalents (mg), Mean ± SD	367.2 ± 9.5	369.5 ± 9.5	364.9 ± 9.0	0.002
Postoperative first leaving bed time (h), M (Q_1_, Q_3_)	10.0 (5.0, 26.0)	20.0 (6.0, 28.0)	6.0 (3.0, 20.0)	< 0.001
Postoperative first exhaust time (h), M (Q_1_, Q_3_)	18.0 (10.0, 26.0)	20.0 (10.0, 29.0)	15.0 (9.5, 25.0)	0.115
Total hospitalization expenses (CNY), Mean ± SD	12,277.7 ± 3385.3	11,718.2 ± 3472.0	12,837.3 ± 3221.3	0.036
Expenses within 24 h (analgesia and adverse reactions) (CNY), Mean ± SD	468.6 ± 60.9	412.8 ± 31.4	524.4 ± 12.6	< 0.001
Expenses within 48 h (analgesia and adverse reactions) (CNY), Mean ± SD	722.6 ± 64.8	669.5 ± 52.2	775.6 ± 5.6	< 0.001

PCIA group: the patients received the PCIA pump alone, TAPB group: the patients received ultrasound-guided TAPB combined with the PCIA pump, *PCIA* patient-controlled intravenous analgesia, *TAPB* transversus abdominis plane block, SD: standard deviation, *CNY* China Yuan

### Cost-effectiveness of Ultrasound-Guided TAPB Combined with PCIA

The ICERs for abdominal wall pain at rest, visceral pain at rest, abdominal wall pain while coughing, and visceral pain while coughing were 16.39, 22.16, 13.31, 13.41 China Yuan (CNY)/VAS-days, respectively, which meant that for every extra point of decrease in the VAS-days score using ultrasound-guided TAPB combined with PCIA, the cost increased by CNY 16.39, 22.16, 13.31, and 13.41, respectively.

### Safety of Ultrasound-Guided TAPB Combined with PCIA

This study also recorded postoperative adverse events including hypotension, bradycardia, respiratory depression, nausea or vomiting, itching, and infection. Regarding the safety of postoperative analgesic techniques, there were no statistically significant differences in postoperative adverse events between the TAPB and PCIA groups (all *P* > 0.05) (Table 3).

**Table 3:** Safety in the PCIA and TAPB groups

Variable	Total (*n* = 160)	PCIA group (*n* = 80)	TAPB group (*n* = 80)	*P*
Hypotension, n (%)				0.497
No	158 (98.8)	80 (100.0)	78 (97.5)	
Yes	2 (1.3)	0 (0.0)	2 (2.5)	
Bradycardia, n (%)				0.210
No	154 (96.3)	79 (98.8)	75 (93.8)	
Yes	6 (3.8)	1 (1.3)	5 (6.3)	
Respiratory depression, n (%)				0.210
No	160 (100.0)	80 (100.0)	80 (100.0)	
Nausea or vomiting, n (%)				1.000
No	130 (81.3)	65 (81.3)	65 (81.3)	
Yes	30 (18.8)	15 (18.8)	15 (18.8)	
Itching, n (%)				1.000
No	159 (99.4)	80 (100.0)	79 (98.8)	
Yes	1 (0.6)	0 (0.0)	1 (1.3)	
Infection, n (%)				1.000
No	159 (99.4)	80 (100.0)	79 (98.8)	
Yes	1 (0.6)	0 (0.0)	1 (1.3)	
Combination therapy, n (%)				0.685
No	130 (81.3)	66 (82.5)	64 (80.0)	
Yes	30 (18.8)	14 (17.5)	16 (20.0)	

PCIA group: the patients received the PCIA pump alone, TAPB group: the patients received ultrasound-guided TAPB combined with the PCIA pump, *PCIA* patient-controlled intravenous analgesia, *TAPB* transversus abdominis plane block

## Discussion

Our study demonstrated the significant efficacy of ultrasound-guided TAPB combined with PCIA on postoperative pain for LC as a part of multimodal analgesia, with lower pain scores (abdominal wall pain or visceral pain) at rest or while coughing and reduced total morphine equivalents within 48 h after LC. In addition, the TAPB group had comparable adverse events to the PCIA group, indicating the safety of ultrasound-guided TAPB combined with PCIA.

Pain is most severe during the first 24 h postoperatively in patients who undergo LC,^19^ and the pain causes are multifactorial: visceral pain due to gall bladder dissection and parietal pain caused by skin incision.^20^ Opioid-based patient-controlled analgesia (PCA) is still commonly used for postoperative pain control and needs to minimize or completely avoid the use of opioids associated with adverse effects whenever possible.^21^ Therefore, adequate postoperative analgesia is necessary to augment the benefits of laparoscopic surgery and optimize patient satisfaction.

In recent years, ultrasound-guided TAPB as an efficient component in multimodal analgesia can provide efficient postoperative analgesia instead of PCIA in LC.^2,22–25^ However, these studies mainly focused on analgesia for abdominal wall pain after TAPB. Few studies have analyzed the analgesic effect of TAPB on visceral pain. The present study evaluated the analgesic effects of TAPB combined with PCIA on abdominal wall pain and visceral pain. We found that TAPB combined with PCIA can provide lower VAS scores (abdominal wall pain and visceral pain) up to 48 h. In addition, reduced analgesic pump compressions and PCIA analgesic dosages within 24 h and decreased total morphine equivalents within 48 h were observed in patients treated with TAPB postoperatively. Therefore, we thought the effect of TAPB combined with PCIA on pain relief was much better than PCIA alone.

Several essential factors influenced the efficacy of postoperative analgesia. On the one hand, the local anesthetic can maintain a fixed concentration and volume for a long time after injecting to improve the analgesic effect. On the other hand, ropivacaine used in TAPB is a long-acting amide drug that mainly blocks the impulse conduction of nerve fibers and relieves pain by blocking the flow of sodium ions into the cell membrane of the nerve fibers. It is one of the ideal medicines for postoperative analgesia, which has the advantages of a long analgesic effect and low toxicity.^26–28^ Of note, the PCIA group did not receive any intraoperative local anesthetic in this study or in clinical practice at our hospital. At other hospitals, the PCIA group may receive intraoperative local anesthesia, either immediately before making surgical incisions or at the termination of a procedure. Thus, studies from other centers are warranted to report intraoperative local anesthesia and the effect of TAPB combined with PCIA versus PCIA alone in these patients.

Recently, a study indicated that PCIA and TAP infiltrations with liposomal bupivacaine were similar in their overall cost-effectiveness strategies to manage postoperative pain.^29^ Therefore, our study further assessed the cost–benefit of TAPB combined with PCIA. As a consequence, postoperative hospital stay, total hospitalization expenses, and expenses within 24 h or 48 h (from analgesia and adverse reactions) were higher in the TAPB group than in the PCIA group. Besides, for every additional point of decrease in the VAS-days score using ultrasound-guided TAPB combined with PCIA, the cost increased by CNY 16.39, 22.16, 13.31, and 13.41, respectively. Nevertheless, patients receiving TAPB combined with PCIA after LC showed more satisfaction.

TAPB is a well-known anesthetic technique that usually blocks the abdominal wall somatic afferent nerves. The blind method based on an anatomical landmark may cause inappropriate obstruction and even injury to the abdominal viscera, such as intestinal puncture and liver injury.^30,31^ In this current study, TAPB with ultrasound guidance was performed after determining the anatomical points; the local structure and needle insertion position were observed in detail to ensure the block’s accuracy and reduce the damage to the peripheral blood vessels. Moreover, the total surgery duration was not significantly different among the groups because the procedure was straightforward and did not consume much time. Additionally, fewer blood vessels and nerves are distributed on the TAP, resulting in the slow clearance of local anesthetics from the TAP and might reduce the risk of systemic toxicity.^26^ Like other studies, we also did not find any complications related to the local drugs, and there were no significant intergroup differences in the side effects.

Despite these merits, there are some limitations to this study. Firstly, we did not make a sensory block assessment to maintain the double-blind study design. However, the exact analgesic effectiveness of TAPB was more reliably reflected by the VAS pain score and analgesic consumption rather than sensory level. Secondly, the TAPB was performed following the induction of general anesthesia, so we could not evaluate parameters at the onset of anesthesia. Furthermore, the doses and volumes of ropivacaine in the TAP were not specified, and clinical signs or symptoms of neurotoxicity were not assessed. Finally, individual variations, including genetic factors that can alter pain sensitivity and analgesic responses, were not considered. Besides, our finding may have limited generalizability. The PCIA group may receive intraoperative local anesthesia, either immediately before making surgical incisions or at the termination of a procedure at other hospitals. Further studies are required to answer these questions.

## Conclusion

Ultrasound-guided TAPB combined with PCIA can provide superior analgesia and decreased total opioid consumption in patients undergoing LC. The observed effectiveness of TAPB combined with PCIA may have a significant role for multimodal pain therapy and have favorable implications for selecting postoperative analgesia after anesthesia.

## Supplementary Information

Below is the link to the electronic supplementary material.

Supplementary file1 (DOCX 41 KB)

Supplementary file2 (PDF 976 KB)

Supplementary file3 (JPG 148 KB)
