# Supplementary material for: Effect of Ultrasound-Guided Transversus Abdominis Plane Block Combined with Patient-Controlled Intravenous Analgesia on Postoperative Analgesia After Laparoscopic Cholecystectomy: a Double-Blind, Randomized Controlled Trial
Source: J Gastrointest Surg. 2022 Sep 13;26(12):2542–50. doi: 10.1007/s11605-022-05450-6 (PMC9674727; doi:10.1007/s11605-022-05450-6)
Supplement: Supplementary file 1 — Supplementary file1 (DOCX 41 KB) [file 11605_2022_5450_MOESM1_ESM.docx]

Supplementary Table 1 VAS and RSS scores at 1 h after LC

| Variable | Total (n=160) | PCIA group (n=80) | TAPB group (n=80) | *P* |
| --- | --- | --- | --- | --- |
| VAS score of abdominal wall pain at rest, n (%) |  |  |  | <0.001 |
| 0 | 2 (1.3) | 2 (2.5) | 0 (0.0) |  |
| 1 | 138 (86.3) | 59 (73.8) | 79 (98.8) |  |
| 2 | 18 (11.3) | 17 (21.3) | 1 (1.3) |  |
| 3 | 2 (1.3) | 2 (2.5) | 0 (0.0) |  |
| VAS score of visceral pain at rest, n (%) |  |  |  | <0.001 |
| 0 | 3 (1.9) | 2 (2.5) | 1 (1.3) |  |
| 1 | 135 (84.4) | 58 (72.5) | 77 (96.3) |  |
| 2 | 20 (12.5) | 18 (22.5) | 2 (2.5) |  |
| 3 | 2 (1.3) | 2 (2.5) | 0 (0.0) |  |
| VAS score of abdominal wall while coughing, n (%) |  |  |  | <0.001 |
| 0 | 1 (0.6) | 1 (1.3) | 0 (0.0) |  |
| 1 | 72 (45.0) | 18 (22.5) | 54 (67.5) |  |
| 2 | 76 (47.5) | 50 (62.5) | 26 (32.5) |  |
| 3 | 11 (6.9) | 11 (13.8) | 0 (0.0) |  |
| VAS score of visceral pain while coughing, n (%) |  |  |  | <0.001 |
| 0 | 2 (1.3) | 1 (1.3) | 1 (1.3) |  |
| 1 | 64 (40.0) | 16 (20.0) | 48 (60.0) |  |
| 2 | 84 (52.5) | 53 (66.3) | 31 (38.8) |  |
| 3 | 10 (6.3) | 10 (12.5) | 0 (0.0) |  |
| RSS score, n (%) |  |  |  | 0.002 |
| 1 | 24 (15.1) | 22 (27.9) | 2 (2.5) |  |
| 2 | 103 (64.8) | 43 (54.4) | 60 (75.0) |  |
| 3 | 19 (12.0) | 8 (10.1) | 11 (13.8) |  |
| 4 | 13 (8.2) | 6 (7.6) | 7 (8.8) |  |

Visual Analogue Scale (VAS): 0 = no pain, 1 = mild pain, 2 = moderate pain, 3 = severe pain; Ramsay sedation scale (RSS): 1 =  anxious and agitated or restless or both, 2 = cooperative, oriented, and tranquil, 3 = responding to commands only, 4 = exhibiting a brisk response to a light glabellar tap.

PCIA group: the patients received the PCIA pump alone, TAPB group: the patients received ultrasound-guided TAPB combined with the PCIA pump, PCIA: patient-controlled intravenous analgesia, TAPB: transversus abdominis plane block, LC: laparoscopic cholecystectomy.

Supplementary Table 2 VAS and RSS scores at 4 h after LC

| Variable | Total (n=160) | PCIA group (n=80) | TAPB group (n=80) | *P* |
| --- | --- | --- | --- | --- |
| VAS score of abdominal wall pain at rest, n (%) |  |  |  | <0.001 |
| 0 | 1 (0.6) | 1 (1.3) | 0 (0.0) |  |
| 1 | 134 (83.8) | 54 (67.5) | 80 (100.0) |  |
| 2 | 25 (15.6) | 25 (31.3) | 0 (0.0) |  |
| VAS score of visceral pain at rest, n (%) |  |  |  | <0.001 |
| 0 | 1 (0.6) | 1 (1.3) | 0 (0.0) |  |
| 1 | 131 (81.9) | 52 (65.0) | 79 (98.8) |  |
| 2 | 28 (17.5) | 27 (33.8) | 1 (1.3) |  |
| VAS score of abdominal wall while coughing, n (%) |  |  |  | <0.001 |
| 1 | 69 (43.1) | 7 (8.8) | 62 (77.5) |  |
| 2 | 85 (53.1) | 67 (83.8) | 18 (22.5) |  |
| 3 | 6 (3.8) | 6 (7.5) | 0 (0.0) |  |
| VAS score of visceral pain while coughing, n (%) |  |  |  | <0.001 |
| 1 | 64 (40.0) | 7 (8.8) | 57 (71.3) |  |
| 2 | 90 (56.3) | 67 (83.8) | 23 (28.8) |  |
| 3 | 6 (3.8) | 6 (7.5) | 0 (0.0) |  |
| RSS score, n (%) |  |  |  | 0.576 |
| 1 | 7 (4.4) | 7 (8.8) | 0 (0.0) |  |
| 2 | 124 (77.5) | 57 (71.3) | 67 (83.8) |  |
| 3 | 12 (7.5) | 7 (8.8) | 5 (6.3) |  |
| 4 | 17 (10.6) | 9 (11.3) | 8 (10.0) |  |

Visual Analogue Scale (VAS): 0 = no pain, 1 = mild pain, 2 = moderate pain, 3 = severe pain; Ramsay sedation scale (RSS): 1 =  anxious and agitated or restless or both, 2 = cooperative, oriented, and tranquil, 3 = responding to commands only, 4 = exhibiting a brisk response to a light glabellar tap.

PCIA group: the patients received the PCIA pump alone, TAPB group: the patients received ultrasound-guided TAPB combined with the PCIA pump, PCIA: patient-controlled intravenous analgesia, TAPB: transversus abdominis plane block, LC: laparoscopic cholecystectomy.

Supplementary Table 3 VAS and RSS scores at 12 h after LC

| Variable | Total (n=160) | PCIA group (n=80) | TAPB group (n=80) | *P* |
| --- | --- | --- | --- | --- |
| VAS score of abdominal wall pain at rest, n (%) |  |  |  | <0.001 |
| 1 | 94 (58.8) | 17 (21.3) | 77 (96.3) |  |
| 2 | 65 (40.6) | 62 (77.5) | 3 (3.8) |  |
| 3 | 1 (0.6) | 1 (1.3) | 0 (0.0) |  |
| VAS score of visceral pain at rest, n (%) |  |  |  | <0.001 |
| 1 | 92 (57.5) | 16 (20.0) | 76 (95.0) |  |
| 2 | 66 (41.3) | 62 (77.5) | 4 (5.0) |  |
| 3 | 2 (1.3) | 2 (2.5) | 0 (0.0) |  |
| VAS score of abdominal wall while coughing, n (%) |  |  |  | <0.001 |
| 0 | 1 (0.6) | 0 (0.0) | 1 (1.3) |  |
| 1 | 56 (35.0) | 5 (6.3) | 51 (63.8) |  |
| 2 | 89 (55.6) | 61 (76.3) | 28 (35.0) |  |
| 3 | 14 (8.8) | 14 (17.5) | 0 (0.0) |  |
| VAS score of visceral pain while coughing, n (%) |  |  |  | <0.001 |
| 0 | 1 (0.6) | 0 (0.0) | 1 (1.3) |  |
| 1 | 53 (33.1) | 5 (6.3) | 48 (60.0) |  |
| 2 | 85 (53.1) | 54 (67.5) | 31 (38.8) |  |
| 3 | 21 (13.1) | 21 (26.3) | 0 (0.0) |  |
| RSS score, n (%) |  |  |  | 1.000 |
| 1 | 3 (1.9) | 2 (2.5) | 1 (1.3) |  |
| 2 | 156 (97.5) | 77 (96.3) | 79 (98.8) |  |
| 3 | 1 (0.6) | 1 (1.3) | 0 (0.0) |  |

Visual Analogue Scale (VAS): 0 = no pain, 1 = mild pain, 2 = moderate pain, 3 = severe pain; Ramsay sedation scale (RSS): 1 =  anxious and agitated or restless or both, 2 = cooperative, oriented, and tranquil, 3 = responding to commands only.

PCIA group: the patients received the PCIA pump alone, TAPB group: the patients received ultrasound-guided TAPB combined with the PCIA pump, PCIA: patient-controlled intravenous analgesia, TAPB: transversus abdominis plane block, LC: laparoscopic cholecystectomy.

Supplementary Table 4 VAS and RSS scores at 24 h after LC

| Variable | Total (n=160) | PCIA group (n=80) | TAPB group (n=80) | *P* |
| --- | --- | --- | --- | --- |
| VAS score of abdominal wall pain at rest, n (%) |  |  |  | <0.001 |
| 0 | 2 (1.3) | 0 (0.0) | 2 (2.5) |  |
| 1 | 125 (78.1) | 48 (60.0) | 77 (96.3) |  |
| 2 | 33 (20.6) | 32 (40.0) | 1 (1.3) |  |
| VAS score of visceral pain at rest, n (%) |  |  |  | <0.001 |
| 0 | 2 (1.3) | 0 (0.0) | 2 (2.5) |  |
| 1 | 124 (77.5) | 47 (58.8) | 77 (96.3) |  |
| 2 | 34 (21.3) | 33 (41.3) | 1 (1.3) |  |
| VAS score of abdominal wall while coughing, n (%) |  |  |  | <0.001 |
| 0 | 1 (0.6) | 0 (0.0) | 1 (1.3) |  |
| 1 | 80 (50.0) | 11 (13.8) | 69 (86.3) |  |
| 2 | 79 (49.4) | 69 (86.3) | 10 (12.5) |  |
| VAS score of visceral pain while coughing, n (%) |  |  |  | <0.001 |
| 0 | 1 (0.6) | 0 (0.0) | 1 (1.3) |  |
| 1 | 79 (49.4) | 11 (13.8) | 68 (85.0) |  |
| 2 | 80 (50.0) | 69 (86.3) | 11 (13.8) |  |
| RSS score, n (%) |  |  |  | 0.323 |
| 2 | 159 (99.4) | 79 (98.8) | 80 (100.0) |  |
| 3 | 1 (0.6) | 1 (1.3) | 0 (0.0) |  |

Visual Analogue Scale (VAS): 0 = no pain, 1 = mild pain, 2 = moderate pain; Ramsay sedation scale (RSS): 2 = cooperative, oriented, and tranquil, 3 = responding to commands only.

PCIA group: the patients received the PCIA pump alone, TAPB group: the patients received ultrasound-guided TAPB combined with the PCIA pump, PCIA: patient-controlled intravenous analgesia, TAPB: transversus abdominis plane block, LC: laparoscopic cholecystectomy.

Supplementary Table 5 VAS and RSS scores at 36 h after LC

| Variable | Total (n=160) | PCIA group (n=80) | TAPB group (n=80) | *P* |
| --- | --- | --- | --- | --- |
| VAS score of abdominal wall pain at rest, n (%) |  |  |  | 0.001 |
| 0 | 2 (1.3) | 0 (0.0) | 2 (2.5) |  |
| 1 | 150 (93.8) | 72 (90.0) | 78 (97.5) |  |
| 2 | 8 (5.0) | 8 (10.0) | 0 (0.0) |  |
| VAS score of visceral pain at rest, n (%) |  |  |  | 0.002 |
| 0 | 1 (0.6) | 0 (0.0) | 1 (1.3) |  |
| 1 | 151 (94.4) | 72 (90.0) | 79 (98.8) |  |
| 2 | 8 (5.0) | 8 (10.0) | 0 (0.0) |  |
| VAS score of abdominal wall while coughing, n (%) |  |  |  | <0.001 |
| 0 | 2 (1.3) | 0 (0.0) | 2 (2.5) |  |
| 1 | 98 (61.3) | 22 (27.5) | 76 (95.0) |  |
| 2 | 60 (37.5) | 58 (72.5) | 2 (2.5) |  |
| VAS score of visceral pain while coughing, n (%) |  |  |  | <0.001 |
| 0 | 1 (0.6) | 0 (0.0) | 1 (1.3) |  |
| 1 | 99 (61.9) | 23 (28.8) | 76 (95.0) |  |
| 2 | 60 (37.5) | 57 (71.3) | 3 (3.8) |  |
| RSS score, n (%) |  |  |  | 0.323 |
| 1 | 1 (0.6) | 0 (0.0) | 1 (1.3) |  |
| 2 | 159 (99.4) | 80 (100.0) | 79 (98.8) |  |

Visual Analogue Scale (VAS): 0 = no pain, 1 = mild pain, 2 = moderate pain; Ramsay sedation scale (RSS): 1 =  anxious and agitated or restless or both, 2 = cooperative, oriented, and tranquil.

PCIA group: the patients received the PCIA pump alone, TAPB group: the patients received ultrasound-guided TAPB combined with the PCIA pump, PCIA: patient-controlled intravenous analgesia, TAPB: transversus abdominis plane block, LC: laparoscopic cholecystectomy.

Supplementary Table 6 VAS and RSS scores at 48 h after LC

| Variable | Total (n=160) | PCIA group (n=80) | TAPB group (n=80) | *P* |
| --- | --- | --- | --- | --- |
| VAS score of abdominal wall pain at rest, n (%) |  |  |  | 0.322 |
| 0 | 3 (1.9) | 1 (1.3) | 2 (2.5) |  |
| 1 | 156 (97.5) | 78 (97.5) | 78 (97.5) |  |
| 2 | 1 (0.6) | 1 (1.3) | 0 (0.0) |  |
| VAS score of visceral pain at rest, n (%) |  |  |  | 0.183 |
| 0 | 3 (1.9) | 1 (1.3) | 2 (2.5) |  |
| 1 | 155 (96.9) | 77 (96.3) | 78 (97.5) |  |
| 2 | 2 (1.3) | 2 (2.5) | 0 (0.0) |  |
| VAS score of abdominal wall while coughing, n (%) |  |  |  | <0.001 |
| 0 | 2 (1.3) | 0 (0.0) | 2 (2.5) |  |
| 1 | 117 (73.1) | 40 (50.0) | 77 (96.3) |  |
| 2 | 41 (25.6) | 40 (50.0) | 1 (1.3) |  |
| VAS score of visceral pain while coughing, n (%) |  |  |  | <0.001 |
| 0 | 2 (1.3) | 0 (0.0) | 2 (2.5) |  |
| 1 | 113 (70.6) | 37 (46.3) | 76 (95.0) |  |
| 2 | 45 (28.1) | 43 (53.8) | 2 (2.5) |  |

Visual Analogue Scale (VAS): 0 = no pain, 1 = mild pain, 2 = moderate pain; Ramsay sedation scale (RSS): all patients having score 2 (2 = cooperative, oriented, and tranquil).

PCIA group: the patients received the PCIA pump alone, TAPB group: the patients received ultrasound-guided TAPB combined with the PCIA pump, PCIA: patient-controlled intravenous analgesia, TAPB: transversus abdominis plane block, LC: laparoscopic cholecystectomy.
