# Supplementary figures and images for: Effect of Ultrasound-Guided Transversus Abdominis Plane Block Combined with Patient-Controlled Intravenous Analgesia on Postoperative Analgesia After Laparoscopic Cholecystectomy: a Double-Blind, Randomized Controlled Trial
Source: J Gastrointest Surg. 2022 Sep 13;26(12):2542–50. doi: 10.1007/s11605-022-05450-6 (PMC9674727; doi:10.1007/s11605-022-05450-6)

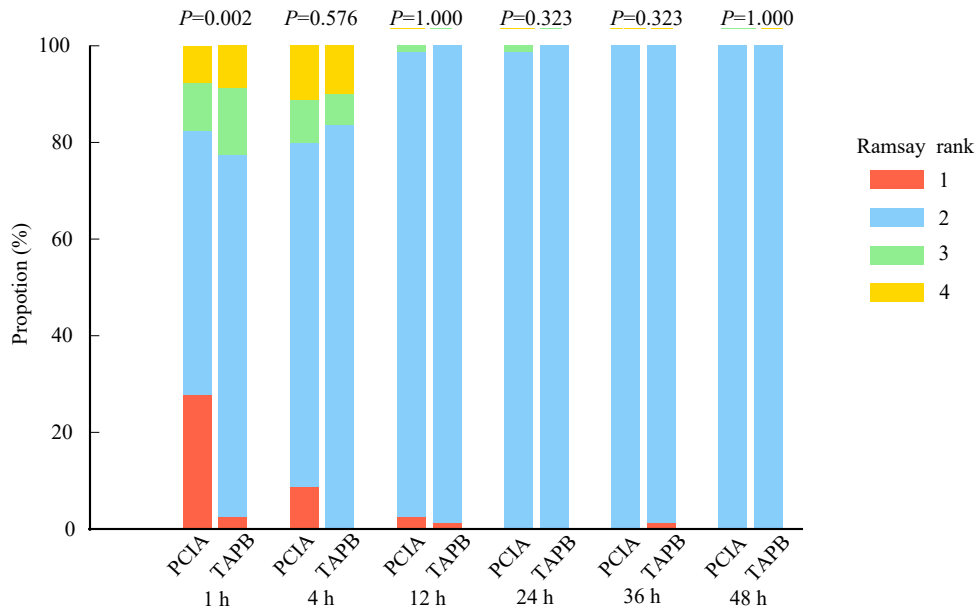

Supplement: Supplementary file 2 — Supplementary file2 (PDF 976 KB) [file 11605_2022_5450_MOESM2_ESM.pdf]

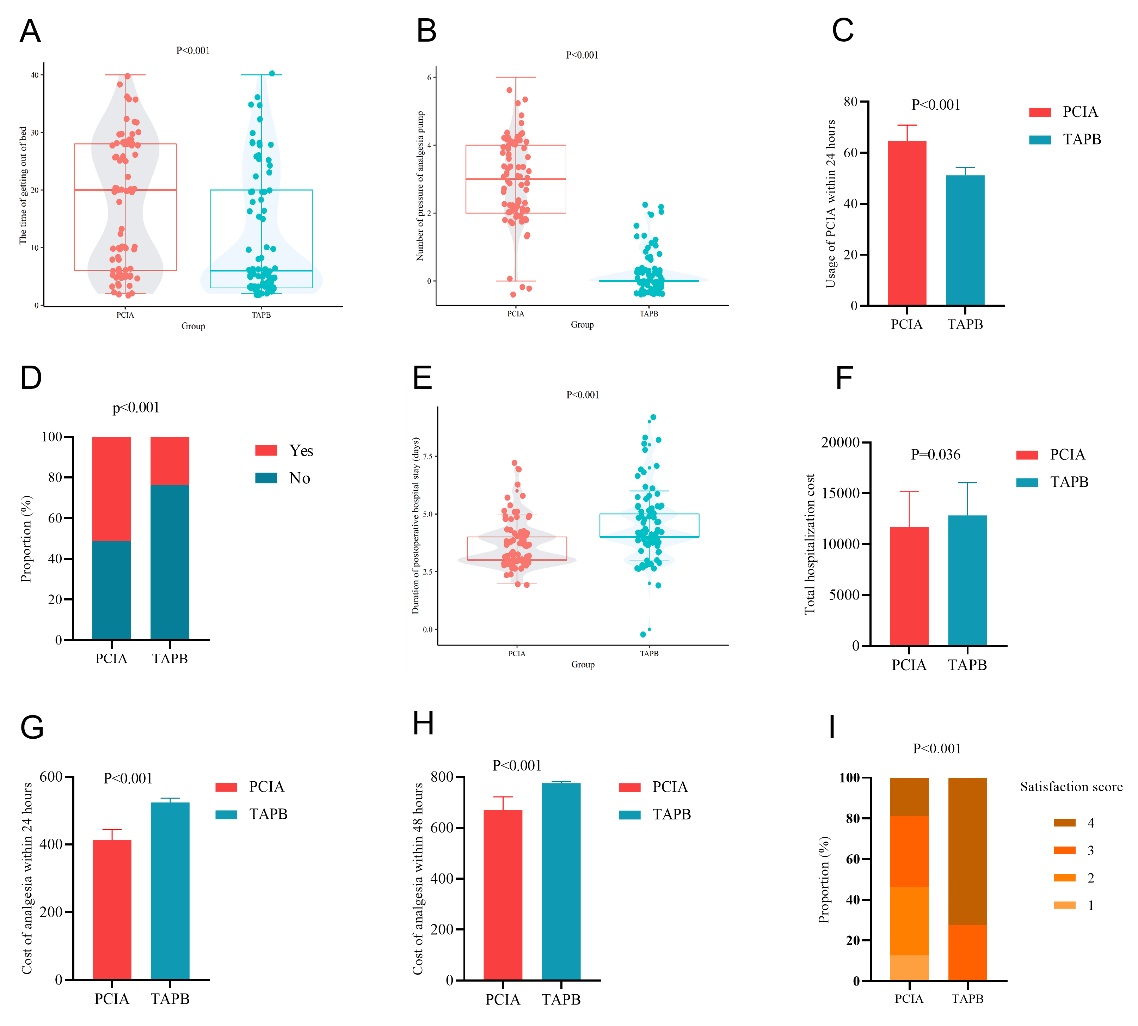

Supplement: Supplementary file 3 — Supplementary file3 (JPG 148 KB) [file 11605_2022_5450_MOESM3_ESM.jpg]
